# Adult pancreatic hemangioma: A case report

**DOI:** 10.3892/ol.2014.2206

**Published:** 2014-06-02

**Authors:** YOSHIKI NAITO, NAOYO NISHIDA, YASUHIRO NAKAMURA, YOSHIKUNI TORII, HIROSHI YOSHIKAI, HIROSHI KAWANO, TETSUJI AKIYAMA, TERUFUMI SAKAI, SATORU TANIWAKI, MASAYA TANAKA, HISASHI KURODA, KOICHI HIGAKI

**Affiliations:** 1 Department of Pathology, St. Mary’s Hospital, Kurume, Fukuoka 830-8543, Japan; 2 Department of Radiology, St. Mary’s Hospital, Kurume, Fukuoka 830-8543, Japan; 3 Department of Gastroenterology, St. Mary’s Hospital, Kurume, Fukuoka 830-8543, Japan; 4 Department of Surgery, St. Mary’s Hospital, Kurume, Fukuoka 830-8543, Japan

**Keywords:** pancreas, pancreatic neoplasm, pancreatic hemangioma

## Abstract

Vascular neoplasms of the pancreas are extremely rare and usually manifest as symptomatic, cystic lesions. This study presents a case that includes the clinicopathologic information used to discriminate pancreatic hemangioma from other types of cystic lesion of the pancreas. A 40-year-old female visited hospital with a chief complaint of abdominal pain. The serum CEA and CA19-9 levels of the patient were within the normal limits. An abdominal computed tomography scan and magnetic resonance imaging showed a 100-mm mass lesion in the body and tail of the pancreas, and the tumor extended toward the retroperitoneum and surrounded the splenic vein. The lesion was subsequently resected. Macroscopically, it was a multiloculated cyst with intracystic hemorrhage. Microscopically, the lesion was composed of numerous, heterogeneous cysts lined by a flattened single layer of cells without significant atypia. Notably, numerous neoplastic vessels extended into the interlobular septa of the pancreas and surrounded the main pancreatic duct. Immunohistochemical analysis showed that the lining cells expressed CD31 and CD34. The lesion was diagnosed as adult pancreatic hemangioma. Surgical treatment may be required when a direct contact between the lesion and the pancreatic tissue is demonstrated using imaging.

## Introduction

Vascular neoplasms usually occur in the visceral organs including the liver, spleen and gastrointestinal tract and occsionaly in the pancreas. A pancreatic vascular neoplasm is an uncommon type of the primary cystic neoplasm (1). The occurrence of pancreatic hemangiomas in adults is extremely rare. Adult pancreatic hemangiomas often manifest as large cystic lesions in middle-age females and in many cases the patients exhibit abdominal pain but no evidence of malignancy (2,3), therefore, accurate diagnosis is essential for their surgical management, using techniques such as pancreatectomy, partial pancreatectomy or non-resection. However, it is often difficult to produce a final differential diagnosis of intraductal papillary mucinous neoplasms, mucinous cystic neoplasms and solid pseudopapillary neoplasms of the pancreas using ultrasound, angiography, computed tomography, or magnetic resonance imaging in preoperative diagnosis due to their fluidity. The present study reports the case of an adult patient with a giant pancreatic hemangioma without recurrence 6 years following curative surgery. Additionally the report discusses the clinicopathological and immunohistochemical data used to distinguish pancreatic hemangiomas from other cystic lesions of the pancreas.

## Case history

### Patient history

**Figure 1:**
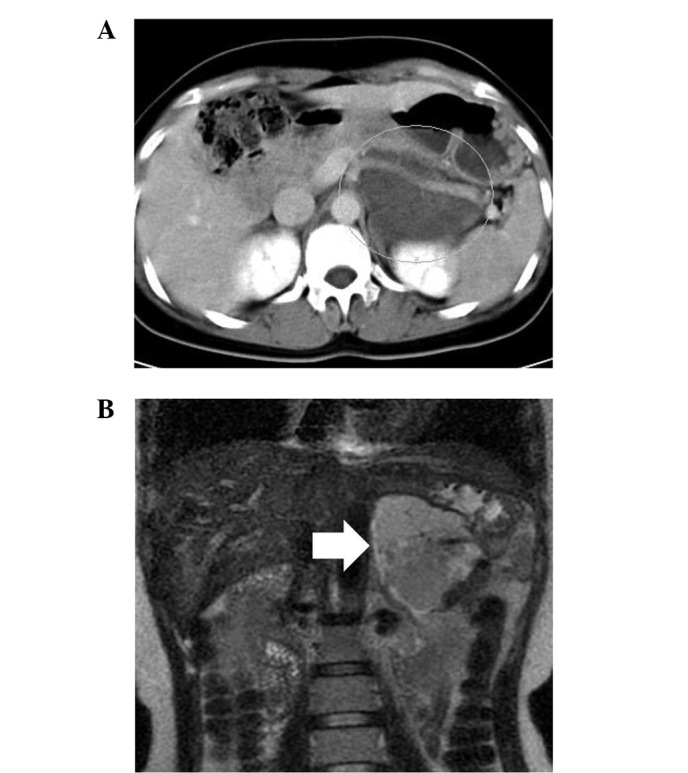
(A) An abdominal computed tomography scan showed a cystic lesion involving the splenic vein, as indicated by the circle. (B) Magnetic resonance imaging identified a high-attenuation, multilocular cyst with septa on T2-weighted imaging, as indicated by the arrow.

A 40-year-old female visited St. Mary’s Hospital (Kurume, Japan) with a chief complaint of abdominal pain. The results of the physical examination were within the normal limits, as were the levels of the two tumor markers, serum CEA and CA19-9. An abdominal computed tomography scan detected a well-defined 100-mm mass with low and high attenuation areas in the body and tail of the pancreas. The tumor extended into the retroperitoneum and surrounded the splenic vein (Fig. 1A). Magnetic resonance imaging showed a high-attenuation, multilocular cystic mass with septa on T2-weighted imaging (Fig. 1B). The case was reviewed in a Multidisciplinary Surgery Conference at St. Mary’s Hospital, and was diagnosed as a cystic neoplasm arising in the retroperitoneum or pancreas.

### Pathological findings

**Figure 2:**
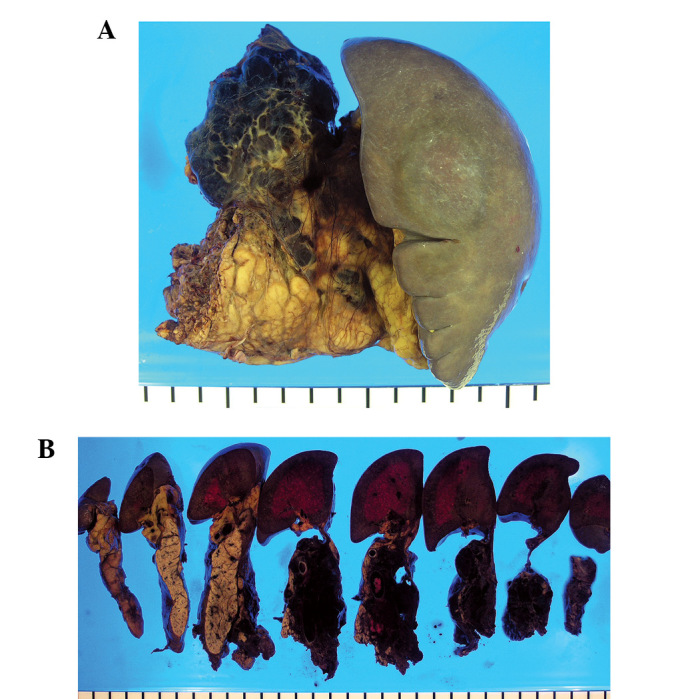
The lesion was (A) a multiloculated cyst with (B) intracystic hemorrhage.

**Figure 3:**
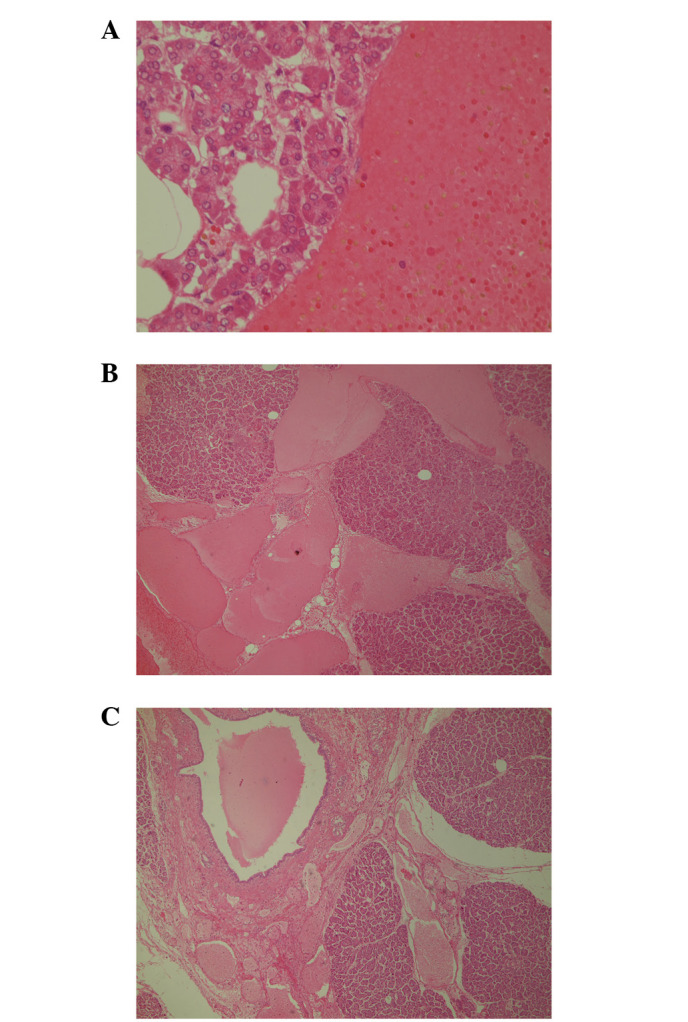
The lesion was composed of (A) numerous cysts lined by a flattened, single layer of cells, and (B) the cysts extended into the interlobular scepta of the pancreas and (C) surrounded the main pancreatic duct. (Magnification, ×40).

**Figure 4:**
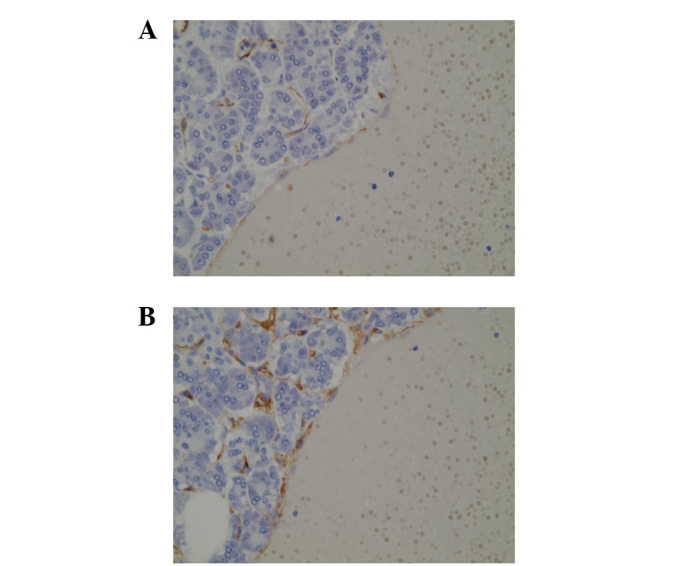
Immunohistochemical findings. The cells were positive for (A) CD31 and (B) CD34 staining. (Magnification, ×400).

Macroscopically, the lesion was multiloculated with intracystic hemorrhage and no mucinous component (Fig. 2). Microscopically, the lesion was composed of numerous and heterogeneous cysts lined by a flattened single layer of cells without significant atypia (Fig. 3A). The cysts extended into the interlobular septa of the pancreas and surrounded the main pancreatic duct (Fig. 3B). Immunohistochemical analysis showed that the cells lining the cysts were expressing CD31 and CD34 (Fig. 4), but were negative for D2–40. Therefore, the lesion was diagnosed as adult pancreatic hemangioma. The background pancreas did not show changes associated with secondary pancreatitis, such as active inflammation or severe atrophic changes of the pancreatic lobules. In addition, the pancreas showed no pancreatic intraepithelial neoplasia (PanIN) lesions. All nodes and margins were negative for neoplasms.

### Treatment and follow up

Following full explanation of the findings, the patient accepted the proposed surgical removal of the tumor. Surgical exploration revealed an 85-mm mass in the pancreatic body and tail that was inseparable from the pancreas. Consequently, a pancreatectomy was performed. The postoperative course was uneventful and six years later, the patient had experienced no recurrence. Patient provided written informed consent.

## Discussion

Pancreatic vascular neoplasm is an uncommon type of primary cystic neoplasm (1). Most pancreatic hemangiomas arise in childhood. Vogel *et al* (3) reported that all the patients with lesions involving the pancreas were <3 years old in a set of 5,051 patients with vascular anomalies. Hemangiomas are reported to proceed in three steps in childhood (4): i) The proliferating phase, which involves a rapid proliferation of the capillaries that lasts up until the age of one year; ii) the involuting phase, in which growth slows and shows inevitable regression until the child is one to five years old; and iii) the involuted phase, whereby improvement continues until the age of 6–12 years, and finally a hemangioma may produce a fibro-fatty residuum by adulthood. Thus, pancreatic hemangioma is a rare disease in adults.

A few adult pancreatic hemangiomas have been reported. As reviewed by Mundinger *et al* in 2009 (2), only nine cases had been reported since 1939 (5–12) and five potential cases were reported prior to 1939. Patients with adult pancreatic hemangiomas are frequently females with a mean age of 55 years, and the hemangiomas are generally large in size, ranging from 3 to 20 mm (2). Most patients with pancreatic hemangioma have abdominal symptoms and a number are severe (2,6–12). Vascular neoplasms in this area are almost benign, even in patients with severe clinical symptoms, and surgical removal of the lesion may be necessary once diagnosed.

The two most common types of cystic neoplasm of the pancreas are the intraductal papillary mucinous neoplasms and mucinous cystic neoplasms of the pancreas, and these neoplasms have malignant potential (1,13,14). However, the adult pancreatic hemangiomas that have been reported since 1939 have shown no malignant potential.

Generally, adult pancreatic hemangiomas are composed of cysts lined by a single layer of uniform endothelial cells that express CD31 and CD34 (2). The case reported in the present study was also composed of numerous cysts lined by neoplastic endothelial cells positive for CD31 and CD34. Therefore, CD31 and CD34 may be useful markers to discriminate between an adult pancreatic hemangioma and other types of cystic neoplasm, particularly cystic lymphangiomas. Notably, in the present case, the neoplastic vessels extended into the interlobular septa of the pancreas and surrounded the main pancreatic duct; however there was no invasion or obstruction of the major pancreatic duct. No changes associated with pancreatitis or precursor lesions of pancreatic cancer, the so-called PanIN lesions, were observed. As an adult pancreatic hemangioma may widely grow along the pancreatic interlobular septa, surgical treatment may be required when imaging shows direct contact between the lesion and the pancreatic tissue.
